# Lercanidipine-Induced Hyponatremia in Elderly Patients: A Not-So-Rare Complication

**DOI:** 10.7759/cureus.85379

**Published:** 2025-06-04

**Authors:** Bashir Mahamud, Katarzyna Mielnik, Naing Myoaung, Marcin Osman, Gideon Mlawa

**Affiliations:** 1 Medical School, Trakia Medical University, Stara Zagora, BGR; 2 Internal Medicine, Queen's Hospital, London, GBR; 3 Internal Medicine/Diabetes and Endocrinology, Barking, Havering and Redbridge University Hospitals National Health Service (NHS) Trust, London, GBR

**Keywords:** adverse drug reaction, calcium channel blockers, elderly, falls, geriatric, hyponatremia, lercanidipine

## Abstract

Hyponatremia, defined as serum sodium concentration below 135 mmol/L, is the most common electrolyte disturbance in the hospitalized geriatric population and is associated with significant morbidity and mortality. While various medications are known to contribute to hyponatremia, calcium channel blockers (CCBs) are not considered. Here we present a case of an elderly patient who developed severe hyponatremia (serum sodium 117 mmol/L). Despite standard interventions in an intensive care setting, the patient's hyponatremia proved resistant to correction. Medication review revealed recent initiation of lercanidipine for hypertension management. Upon discontinuation of lercanidipine, the patient's hyponatremia and confusion resolved. Current NHS guidelines do not recommend routine electrolyte monitoring when prescribing CCBs, unlike recommendations for ACE inhibitors and ARBs. We recommend implementing routine electrolyte monitoring when initiating CCBs in this vulnerable population group to prevent potentially life-threatening complications.

## Introduction

Life expectancy in the UK has steadily increased by about 2.5 years per decade. However, this has also led to a rise in the time people spend in poor health. Aging is a complex process that leads to so-called geriatric syndrome, a combination of issues such as impaired mobility, incontinence, cognitive decline, and frailty [1]. As individuals age, they often develop multiple chronic conditions, making them more vulnerable to complications of different medications [1-2].

Falls are a common occurrence among the elderly, with estimates suggesting that one in three individuals over 65 living in the community experiences at least one fall per year [3,4]. Falls in older adults can lead to significant morbidity, fragility fractures, and loss of independence, contributing to physical and mental deterioration. Given their multifactorial nature, falls require a comprehensive assessment to identify underlying causes [5]. 

Hyponatremia is a frequent electrolyte imbalance in older adults and is known to increase the risk of falls and frailty. Studies suggest that in geriatric and frail patients, hyponatremia often has multiple contributing factors, including endocrine disorders such as syndrome of inappropriate antidiuretic hormone secretion (SIADH), "tea and toast" syndrome, and medication use [1,6-8].

Older adults take multiple medications, with drugs like diuretics, selective serotonin reuptake inhibitors (SSRIs), and antidepressants increasing the risk of hyponatremia [9]. Long-acting dihydropyridine calcium channel blockers (CCBs), such as amlodipine and lercanidipine, are widely prescribed for hypertension in older patients. These medications are considered safe and are often used in patients with conditions like diabetes, chronic lung disease, and peripheral vascular disease. However, they are not without risks [9,10]. It has been shown that when a patient has been started on a CCB, they are at higher risk of hospitalization with hyponatremia. This effect is not seen once established on the medication [11]. Here, we present a case of lercanidipine-induced hyponatremia in an elderly gentleman.

## Case presentation

An 80-year-old man was admitted to the hospital following a fall, which resulted in a traumatic head injury. Prior to the fall, he had been feeling unwell and lethargic for the past week. His medical background included transient ischemic attack (TIA), stage 3 chronic kidney disease (CKD), prediabetes, hypertension, obstructive sleep apnea, and benign prostatic hyperplasia. Functional status lives with family in a house, indoor mobility unaided, outdoor, able to walk short distances up to ten meters. Uses a wheelchair for longer distances. Independent in personal care and transfers. Family helps with domestic activities. No previous fall history or previous hyponatremia.

Before admission, his regular medications included atorvastatin, clopidogrel, lansoprazole, loratadine, allopurinol, prochlorperazine, solifenacin, and lercanidipine. The patient was started on lecardipine for hypertension two weeks before admission. His Rockwood Clinical Frailty Score was 3 before admission.

**Figure 1:**
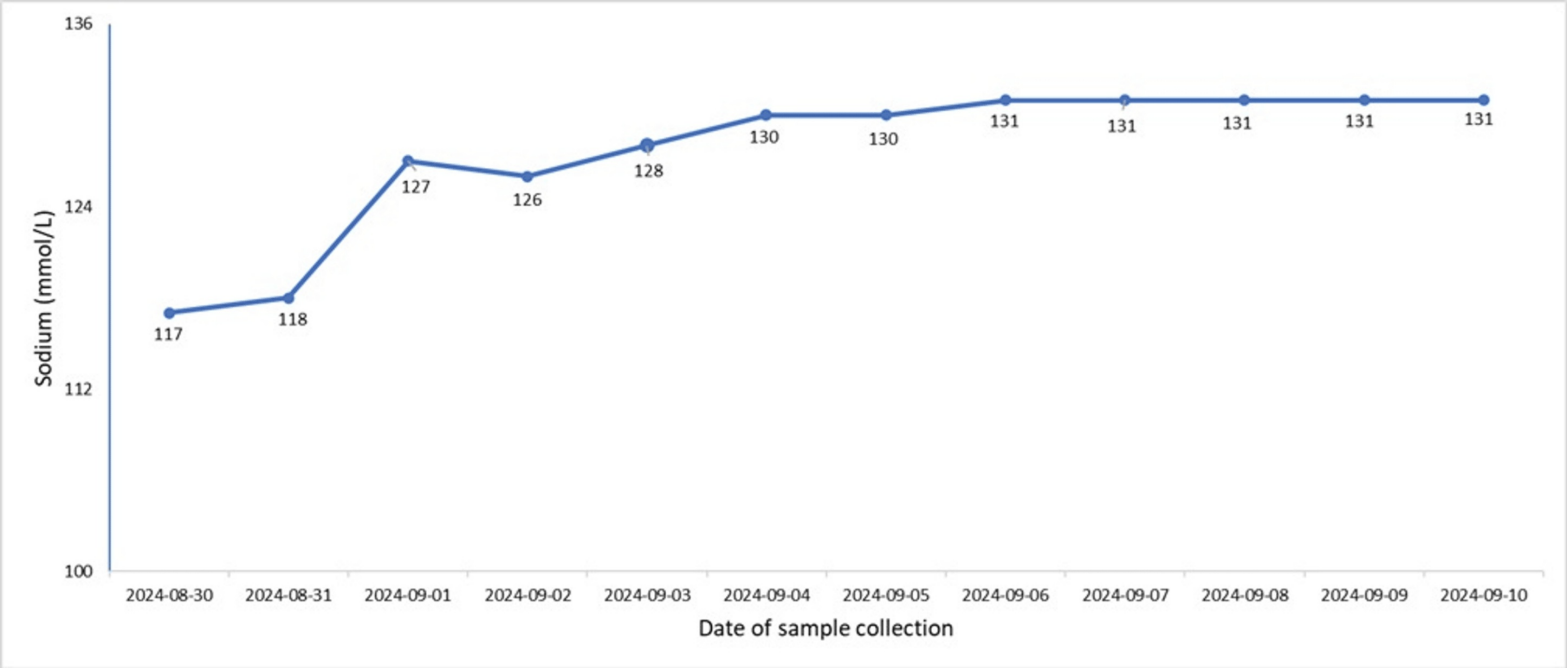
A graphic trend of patient sodium levels The graph highlights the correction of hyponatremia. Lecardipine was stopped on 30/08/2025.

On arrival, his Glasgow Coma Scale (GCS) score was 15/15. His vital signs revealed mild tachycardia (heart rate of 105 bpm, sinus rhythm) and a blood pressure of 170/89 mmHg. There were no obvious injuries on examination. A full-body trauma CT scan was performed, showing no evidence of fractures, haemorrhage, or infection. His initial blood results showed severe hyponatremia, with a sodium level of 117 mmol/L (Table 1 and Figure 1). His thyroid function, cortisol levels, N-terminal pro B-type natriuretic peptide (NT-proBNP), and vitamin B12 levels were all within normal limits. Given the severity of his hyponatremia, he was admitted to intensive care for close monitoring and management.

**Table 1:** Shows patient blood test results, indicating severe hyponatremia

Investigation	Result	Normal Reference	Units
Sodium	117	133-146	mmol/L
Potassium	4.6	3.5-5.3	mmol/L
Urea	4.6	2.5-5.3	mmol/L
Creatinine	130	60-104	umol/L
C-reactive Protein (CRP)	1	<5	mg/L
Thyroid Stimulating Hormone (TSH)	1.92	0.27-4.2	mU/L
Hemoglobin (Hb)A1C	45	<42	nmol/mol
Cortisol	360	-	nmol/L
Urine Osmolality	413	-	mmol/kg
Serum Osmolality	258	275-295	mmol/kg
Urine Sodium	54	-	mmol/L

**Figure 2:**
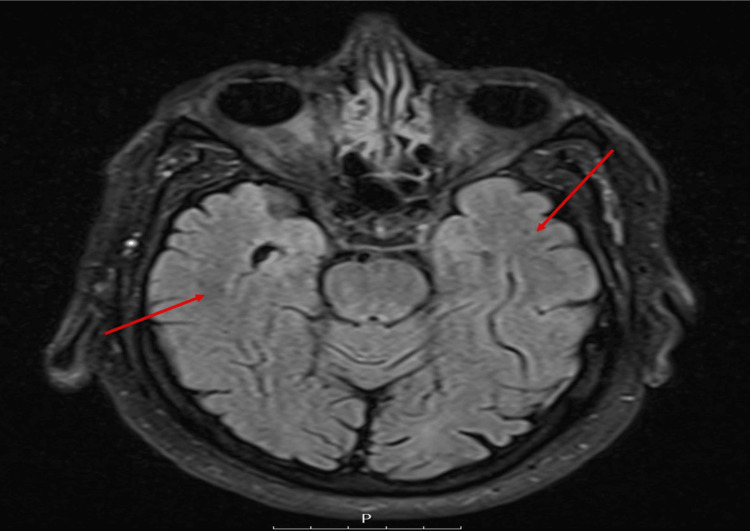
MRI brain image showing age related scattered small vessel disease

Subsequent hyponatremia screens showed that his serum osmolality was 258 mmol/kg, urine osmolality was 413 mmol/kg, and urine sodium was 54 mmol/L (Table 1). An MRI brain scan (Figure 2) performed to investigate the cause of the hyponatremia showed age-related scattered small vessel disease but no acute abnormalities.

Whilst in ICU, the patient was treated with slow intravenous (IV) 0.9% sodium chloride and placed on fluid restriction (1.5 L per 24 hours) to ensure a gradual sodium correction of no more than 8mmol/L per day. Despite these interventions, his hyponatremia did not resolve. A suspicion of medication-induced hyponatremia, lercanidipine was discontinued on 31/08/2024, and this resulted in marked improvement of patient sodium levels (Figure 1).

## Discussion

Hyponatremia is one of the most common electrolyte disturbances encountered in hospitalized older adults and is linked to increased morbidity. Hyponatremia is defined as a serum sodium concentration below 135 mmol/L. Even mild hyponatremia has been associated with an increased risk of fractures, osteoporosis, and death [12,13]. In elderly patients, hyponatremia has been implicated in recurrent falls, due to its effects on gait and attention. Falls in this age group often lead to longer hospital stays, increased risk of rehabilitation placement, and a greater likelihood of further falls and loss of independence [5]. The cause of hyponatremia in frail older adults is often multifactorial, with polypharmacy being a major contributing factor. Studies have indicated that hospitalized elderly patients frequently had hyponatremia, with polypharmacy being highly prevalent among geriatric patients. Common medications such as diuretics, antidepressants, antihypertensives, and antiepileptics are well-established causes of both asymptomatic and symptomatic hyponatremia [14,15].

Lercanidipine is a third-generation dihydropyridine CCB with high lipophilicity, allowing for prolonged smooth muscle relaxation and vasodilation. It exerts its antihypertensive effects by competitively binding to L-type calcium channels on peripheral and coronary arteries. Compared to other CCBs, lercanidipine has been shown to lower blood pressure more effectively in elderly patients with fewer adverse effects [11,15,16]. Despite its renal protective effects, CCBs like lercanidipine have natriuretic and diuretic properties that can increase urinary sodium excretion by up to fourfold. This characteristic can potentially lead to hyponatremia, as seen in the case presented here [10,15]. There is limited literature available on the potential side effects of these regularly prescribed medications.

Tun et al. reported the case of a patient whose hypertension was managed with amlodipine and who developed hyponatremia. After they excluded other causes of his hyponatremia, amlodipine was substituted with bisoprolol, and his sodium levels normalised. In the case presented here, the patient reported developing significant lethargy, which culminated in a traumatic fall. On admission, he was severely hyponatremic with a serum sodium value of 117 mmol/L (Table 1 and Figure 1). He had serum osmolality of 258 mmol/kg, urine osmolality of 413 mmol/kg, and urinary sodium of 61 mmol/L (Table 1). Despite careful correction of hyponatremia in the intensive care setting with fluid, there was limited improvement in his sodium levels. The case was discussed with the local endocrine team, who advised fluid restriction and checking thyroid function and 9 am cortisol, all of which were within normal levels (Table 1). Whilst admitted, the patient became increasingly more confused, as such he had an MRI of the brain which demonstrated only age-related scattered small vessel disease but no acute abnormalities (Figure 2). Upon review of his medication, it was identified that prior to admission, he was started on lercanidipine for a new diagnosis of hypertension by his general practitioner, and this was discontinued, and there was a reversal of both his acute hyponatremia and confusion.

Given how widely CCBs are prescribed in primary care, rare adverse effects such as hyponatremia may not be fully appreciated in clinical practice. Currently, monitoring for hyponatremia is not a standard practice when prescribing CCBs, particularly in the community setting where hypertension management often begins. A study by Falhammar et al. highlighted that newly initiated CCBs, beta-blockers, angiotensin-converting enzyme (ACE) inhibitors, and angiotensin receptor blockers (ARBs) carry a high risk of hospitalization due to hyponatremia, particularly in the elderly [11]. These findings highlight the important need for monitoring electrolyte levels when starting older adults on CCBs. Notably, NICE guidelines do not currently recommend routine electrolyte monitoring for patients commenced on CCBs, unlike ACE inhibitors and ARBs, which require strict monitoring of patient renal function and electrolytes.

## Conclusions

Elderly patients often have multiple comorbidities and high medication burden, which makes them susceptible to many adverse effects, including electrolyte imbalances. Lercanidipine and other CCBs are recommended treatment for hypertension in this age group by NICE; however, side effects like hyponatremia are often not monitored. The case reported here highlights the importance of considering CCB-induced hyponatremia, particularly in elderly patients presenting with falls or confusion. We recommend that routine electrolyte monitoring be considered when starting CCBs in elderly patients to prevent potentially life-threatening complications, such as medication-induced hyponatremia.
